# Opium in Cancer of the Uterus

**Published:** 1901-03-23

**Authors:** 


### OPIUM IN CANCER OF THE UTERUS.

Dr. Gerald Leighton[1] asks a question which all of us have had to ask ourselves many a time, namely, "Given a case of cancer where surgical treatment is impossible, what treatment offers the best means of making the patient's life bearable as long as it lasts?" And he answers it as more than one observer has answered it before by saying, "Turn the patient into an unconscious opium eater." He describes a case of cancer of the cervix uteri in a widow, aged 71 years, in which it was decided, after consultation, that operation would be inadvisable. He tells how, under the influence of pain, the patient's health gave way; and then he goes on to show how great an improvement in her condition resulted from the gradual and progressive administration of opium, given in sufficient doses to ensure sleep and entire freedom from pain for six or eight hours at a stretch. Under this she soon began to show a marked improvement in her general condition, took food well, and instead of dying, as had been expected a few weeks before, began to get out in her carriage a little. The dose of opium, however, had to be constantly increased to secure this result, so that in three months' time she was taking 3 oz., and in another month 4 oz. of tincture of opium daily. After a time tincture of belladonna was added with apparent benefit. To sum up, the patient had lived two years longer than had been at first thought probable, and the last 18 months of her life had been incomparably more comfortable than were the first 12 months after the disease showed itself. Throughout the whole of the last two years there was no difficulty with the bowels, there were no head symptoms, and a plain milk diet was well taken. In the end she was taking over 12 oz. of tincture of opium daily. But she never knew that she was taking any.

The use of opium in inoperable cancer has been advocated by many authorities, and not merely as a means of relieving pain, but with the object of modifying and restraining the cancer process. Dr. Herbert Snow has especially urged the utility of opium, holding that its use has a considerable influence in preventing recurrence, even where from the condition of the parts at the time of operation such a result is feared. Writing in 1896 he said that he had gradually learned to combine cocaine with the opium, and, he says, with generally better results than he would have expected from opium alone. He mentions a considerable number of cases in which the disease, to say the least of it, advanced very slowly under the use of one half to one grain of cocaine three times a day combined with opium. The doses he mentions, however, are far smaller than those given by Dr. Leighton.

There is but little doubt that many surgeons tend to hold back from giving opium in cases of cancer, hoping probably to keep it in reserve so as to give the unfortunate patients the benefit of its full activity when at last its employment becomes absolutely necessary; but it may fairly be questioned whether this course is not somewhat of a mistake. There is much reason to believe that opium is something more than a palliative, and that it does exercise some inhibitory influence over the cancer process, as indeed over all cell growth, while one may very justifiably hold, what most sufferers will tell us is the truth, namely, that nothing is worse than pain—not merely because it is bad to bear, but because of its injurious influence upon every function, both of mind and body.

